# Downregulation of ATG5-dependent macroautophagy by chaperone-mediated autophagy promotes breast cancer cell metastasis

**DOI:** 10.1038/s41598-017-04994-x

**Published:** 2017-07-06

**Authors:** Qi Han, Youcai Deng, Sha Chen, Rui Chen, Mingzhen Yang, Zhujun Zhang, Xiongshan Sun, Wei Wang, Ying He, Fangjie Wang, Xiaodong Pan, Peng Li, Wenjing Lai, Hongqin Luo, Pei Huang, Xiao Guan, Yafei Deng, Jun Yan, Xianjie Xu, Yan Wen, An Chen, Chuanmin Hu, Xiaohui Li, Shuhui Li

**Affiliations:** 1 Department of Clinical Biochemistry, Faculty of Medical Laboratory Science, Southwest Hospital, Third Military Medical University, Chongqing, 400038 China; 2 Institute of Materia Medica, College of Pharmacy, Third Military Medical University, Chongqing, 400038 China; 3 Institute of Hepatobiliary Surgery, Southwest Hospital, Third Military Medical University, Chongqing, 400038 China

## Abstract

Recent data have shown that the expression of lysosome-associated membrane protein type 2 A (LAMP2A), the key protein in the chaperone-mediated autophagy (CMA) pathway, is elevated in breast tumor tissues. However, the exact effects and mechanisms of CMA during breast cancer metastasis remain largely unknown. In this study, we found that the LAMP2A protein level was significantly elevated in human breast cancer tissues, particularly in metastatic carcinoma. The increased LAMP2A level was also positively correlated with the histologic grade of ductal breast cancer. High LAMP2A levels also predicted shorter overall survival of breast cancer patients. Downregulation of CMA activity by LAMP2A knockdown significantly inhibited the growth and metastasis of both MDA-MB-231 and MDA-MB-468 breast cancer cells *in vivo* and *in vitro*, while upregulation of CMA activity by LAMP2A overexpression had the opposite effect. Mechanistically, we found that elevated CMA activity mediated increased growth and metastasis of human breast cancer cells by downregulating the activity of autophagy-related gene 5 (ATG5)-dependent macroautophagy. Collectively, these results indicate that the anti-macroautophagic property is a key feature of CMA-mediated tumorigenesis and metastasis and may, in some contexts, serve as an attractive target for breast cancer therapies.

## Introduction

Breast cancer is an important worldwide health problem. Metastasis remains the primary cause of death for patients with breast cancer. Approximately 30% of patients develop metastasis or recurrence, even when diagnosed at an early stage of breast cancer development^1, 2^. However, the molecular mechanisms that govern metastatic dissemination are poorly understood.

Autophagy is a primary pathway by which intracellular components, such as proteins and organelles, are delivered to lysosomes and degraded, and this process plays a crucial role in tumor initiation and progression^3^. Chaperone-mediated autophagy (CMA) and macroautophagy are the two best-characterized pathways in mammalian cells^4, 5^. CMA possesses the unique characteristics of selectivity, saturability and competitivity because it targets and degrades specific soluble proteins that contain a recognizable peptide sequence motif (KFERQ)^6^. LAMP2A is the principal limiting component of the CMA pathway, as its protein levels at the lysosomal membrane directly determine CMA activity^7, 8^.

It has been demonstrated that CMA plays an important role in many human diseases, such as neurodegenerative diseases, metabolic disorders, and liver diseases^9–11^. Recently, there has been growing interest in elucidating the role of CMA in cancer pathogenesis and finding new treatments based on CMA activity modulation. Kon *et al*. reported that CMA was required for tumor growth and metastasis in lung cancer^12^. Another study reported that elevated LAMP2A expression was observed in ductal carcinoma tissues from 7 breast cancer patients^13^. However, the role of CMA activity in the malignant progression and metastasis of breast cancer remains largely unknown.

Recent studies have shown that cross-talk between CMA and macroautophagy exists in many untransformed cell types and even *in vivo* ^14–16^. However, to date, the interconnections between macroautophagy and CMA pathways in breast cancer cells remain unclear. Given the evidence that the activity of the ATG5-ATG12 complex was upregulated in LAMP2A knockdown HeLa cells^17^ and that macroautophagy serves as a pro-death mechanism in breast cancer cells^18–20^, we hypothesized that the regulation of breast cancer cell metastasis by CMA may be associated with ATG5-dependent macroautophagy.

## Results

### CMA activity is elevated in primary human breast cancer

**Figure 1:**
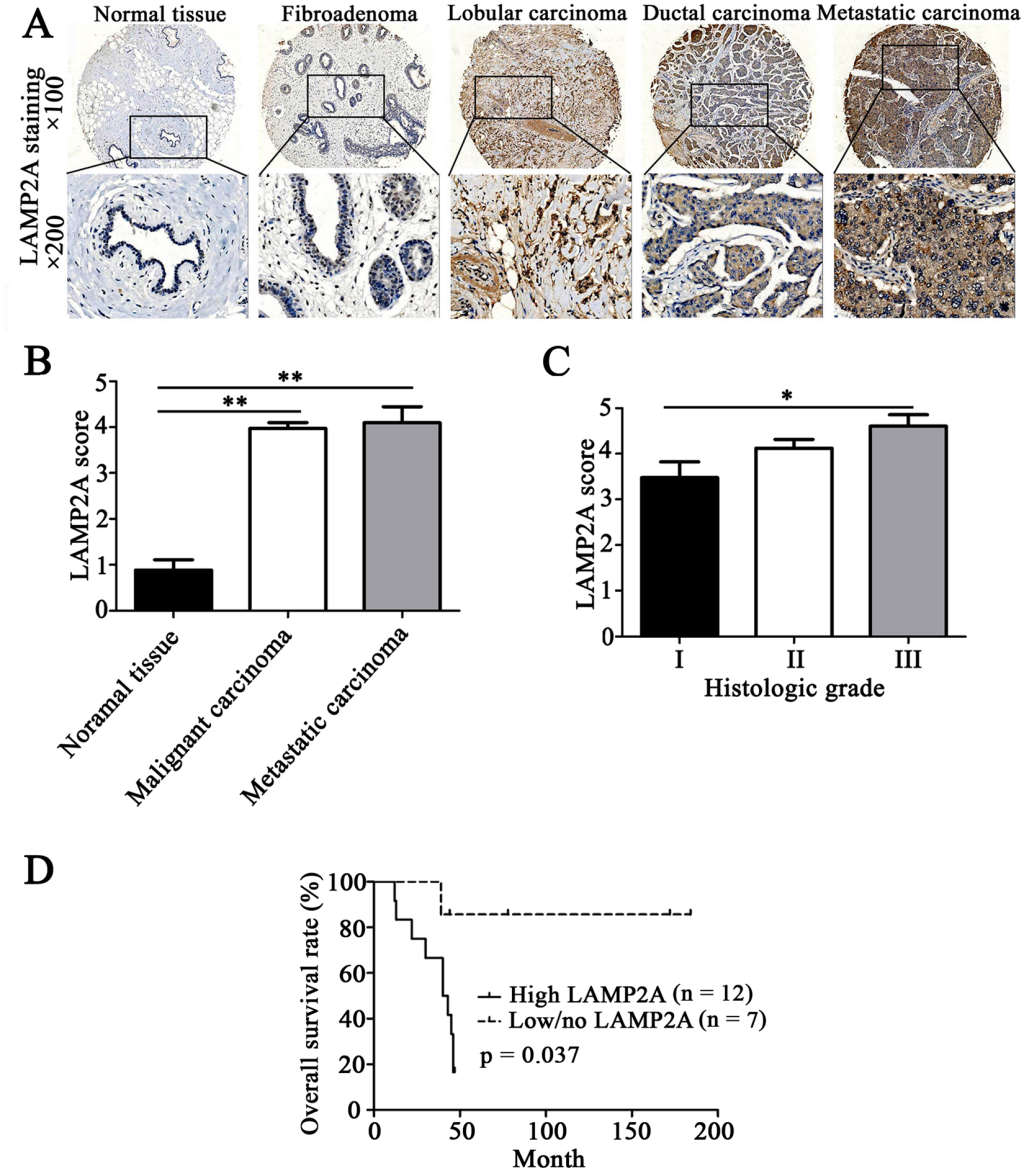
CMA activity is elevated in human breast cancer. (**A**) Immunostaining for LAMP2A in normal breast epithelium tissues, fibroadenoma, breast cancer and metastatic lymph node tumor tissues (100× and 200×). (**B**) Immunoreactivity scores of LAMP2A levels in normal tissues, malignant carcinoma and metastatic carcinoma. (**C**) Immunoreactivity scores of LAMP2A in tumors of different histologic grades. Among all the invasive ductal carcinoma tumors, 21 were Grade I, 74 were Grade II, and 28 were Grade III. (**D**) Kaplan–Meier plots of LAMP2A expression in 19 cases of breast cancer patients. Overall survival rate was determined by the log-rank test. Each sample was assigned an immunoreactivity score that calculated the sum of the intensity of positive tumor cells (0 = none; 1 = weak; 2 = intermediate, 3 = strong) and the estimated fraction of positive staining tumor cells (0 = none, 1 ≤ 10%, 2 = 10–50% and 3 ≥ 50%) ranging from 0 and 2–6. We identified TMA scoring >3 as LAMP2A-positive tumors and TMA scoring ≤3 as LAMP2A-negative tumors (*P < 0.05, **P < 0.01).

To determine whether CMA activity is increased in breast tumors or the related metastatic carcinoma in lymph nodes, we assessed LAMP2A expression in 166 breast cancer specimens and 21 normal breast epithelium specimens by immunohistochemical staining. LAMP2A protein expression showed a statistically significant difference between malignant breast cancer tissues, metastatic carcinoma of the lymph nodes and normal tissues. Its expression level was higher in the malignant breast cancer tissues and metastatic carcinoma than that in the normal breast epithelium tissues (Fig. 1A). The immunoreactivity score for CMA activity (calculated as the sum of the LAMP2A intensity and percentage scores) in metastatic carcinoma patients was significantly higher (~4.0-fold) than that in the normal tissue group (*P* < 0.01, Fig. 1B). Further analysis revealed that LAMP2A protein expression was also positively correlated with the histologic grade of the ductal carcinoma, and LAMP2A expression was significantly higher in grade III than in grade I breast tumors (*P* < 0.05, Fig. 1C). Histologic grade is associated with the outcome of breast cancer therapy and the survival rate of breast cancer patients. We also found that increased LAMP2A expression was correlated with the overall survival of patients; the overall survival rate of patients with low LAMP2A expression was far higher than that of patients with high LAMP2A expression in the long term (*P* < 0.05, Fig. 1D). These results demonstrated that LAMP2A-positive tumors exhibited more malignancy than LAMP2A-negative tumors, which suggested that a high level of CMA activity may contribute to the tumorigenesis and metastasis of breast cancer.

### CMA promotes breast cancer cell growth and survival *in vitro* and *in vivo*

**Figure 2:**
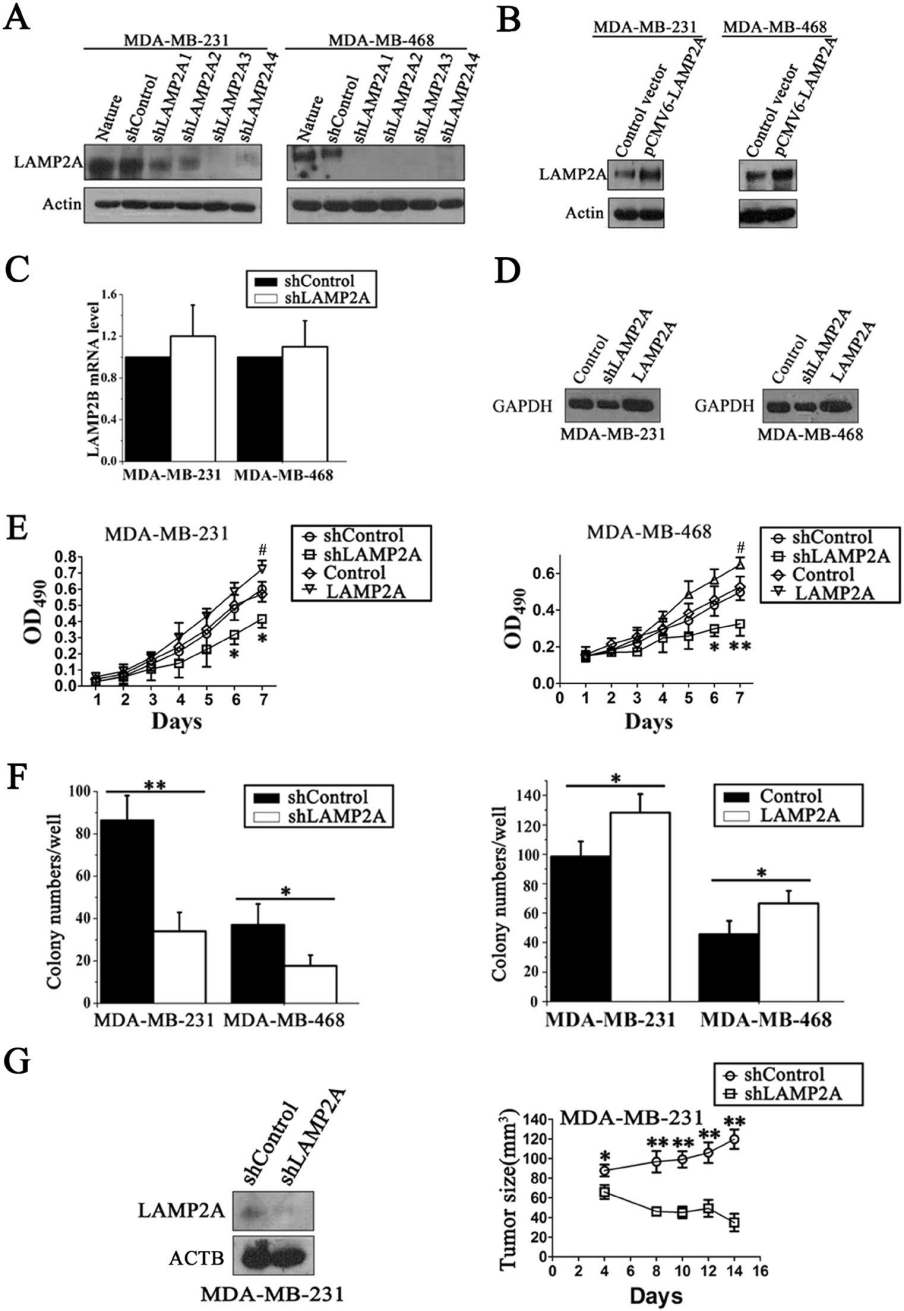
CMA promotes breast cancer cell growth and survival. (**A**) The stable inhibitory efficiency of shRNAs against *LAMP2A* in MDA-MB-231 and MDA-MB-468 cells was detected. (**B**) Western blotting detected LAMP2A overexpression by transient transfection in two breast cancer cell lines. (**C**) The *LAMP2B* mRNA level was determined in MDA-MB-231 and MDA-MB-468 cells by qRT-PCR. (**D**) Purified GAPDH protein was incubated with intact lysosomes isolated from the stable LAMP2A knockdown and overexpression cells and then harvested, fractionated and immunoblotted with a GAPDH antibody. (**E**) The cell growth rate was determined by an MTT assay at indicated timepoints in MDA-MB-231 cells and MDA-MB-468 cells. (**F**) A colony formation assay was performed to detect the proliferative capability of the breast cancer cells. The cells were seeded onto 6-well plates and allowed to form colonies for two weeks. All values are expressed as the mean ± SD of three different experiments; *P < 0.05 and **P < 0.01, shLAMP2A versus shControl at the same timepoint; ^#^ *P* < 0.05, LAMP2A overexpression group versus the control group at the same timepoint. (**G**) 1 × 10^7^ shLAMP2A or shControl MDA-MB-231 cells were subcutaneously injected into nude mice. The LAMP2A protein levels in the tumors at the time of resection were determined by immunoblots (left), and the size of the tumors was monitored by the standard formula length × width × width × 0.5 (right) (n = 5; *P < 0.05, shLAMP2A or shControl).

Altering the expression levels of LAMP2A is the most efficient way to regulate CMA activity^15^. To determine whether CMA activation in breast cancer cells is required for their oncogenic behavior, two human breast cancer cell lines with different metastatic potentials (highly invasive MDA-MB-231 cells and less invasive MDA-MB-468 cells)^21^ were tested by manipulating the CMA activity. We downregulated or upregulated CMA activity in these two human breast cancer cell lines using lentiviral-mediated knockdown of LAMP2A or a LAMP2A overexpression vector, respectively (Fig. 2A,B). qRT-PCR analysis indicated that LAMP2A shRNA1 showed no off-target effects on LAMP2B (Fig. 2C).

When CMA is activated, the lysosomes active for CMA will relocate to the perinuclear region, and CMA activity can be monitored indirectly by investigating the distance between LAMP2A-positive lysosomes and the nucleus by immunostaining cells with both a LAMP2A antibody and LysoTracker^7, 22^. The increased distance of LAMP2A-positive lysosomes from the nucleus shown by immunofluorescence suggested that knockdown of LAMP2A in MDA-MB-231 and MDA-MB-468 cells suppressed CMA activity, while LAMP2A overexpression showed the opposite effect (Supplementary Fig. S1A and B). To observe CMA activity more directly, we used a classic method of monitoring CMA activity by measuring the association of purified GAPDH with active intact lysosomes^13^. The purity of the lysosome preparation was detected by the enrichment of the lysosomal marker LAMP2A, a marked decrease of the mitochondria marker HSP60 and downregulation of the cytoplasmic marker β-actin^23^ (Supplementary Fig. S1C). The integrity of lysosomal membranes after isolation was validated by β-hexosaminidase activity^23^ (Supplementary Fig. S1D). A decreased lysosomal association of GAPDH was found after knockdown of LAMP2A in both MDA-MB-231 and MDA-MB-468 cells, whereas overexpression of LAMP2A in these two cell lines showed the opposite effect (Fig. 2D).

We further explored the effects of CMA on breast cancer cell growth and survival with an MTT-based cell viability assay and a colony formation assay. Through knockdown or overexpression of LAMP2A in MDA-MB-231 and MDA-MB-468 breast cancer cells, we found that LAMP2A levels positively regulated the growth and survival of these cells (Fig. 2E,F). We also used another *LAMP2A* shRNA (shLAMP2A2) to confirm the effect of CMA on cell growth in MDA-MB-231 cells, which showed a similar tendency (Supplementary Fig. S2A). However, this phenomenon did not exist in normal breast epithelial MCF10A cells (Supplementary Fig. S3A and B).

To further explore whether CMA activation is essential for xenograft tumor growth *in vivo*, we first analyzed the effect of CMA on the tumorigenic capability of human breast cancer cells in nude mice. Nude mice were inoculated with shLAMP2A or shControl MDA-MB-231 breast cancer cells via subcutaneous injection, and the tumor size was quantified every 2 days until day 16. The tumor size in the shLAMP2A group was significantly smaller than that in the control group (Fig. 2G). Thus, the above results demonstrated that CMA promotes the growth and survival of breast cancer cells, but not of normal breast epithelial cells.

### CMA promotes breast cancer cell migration and invasion *in vitro* and *in vivo*

**Figure 3:**
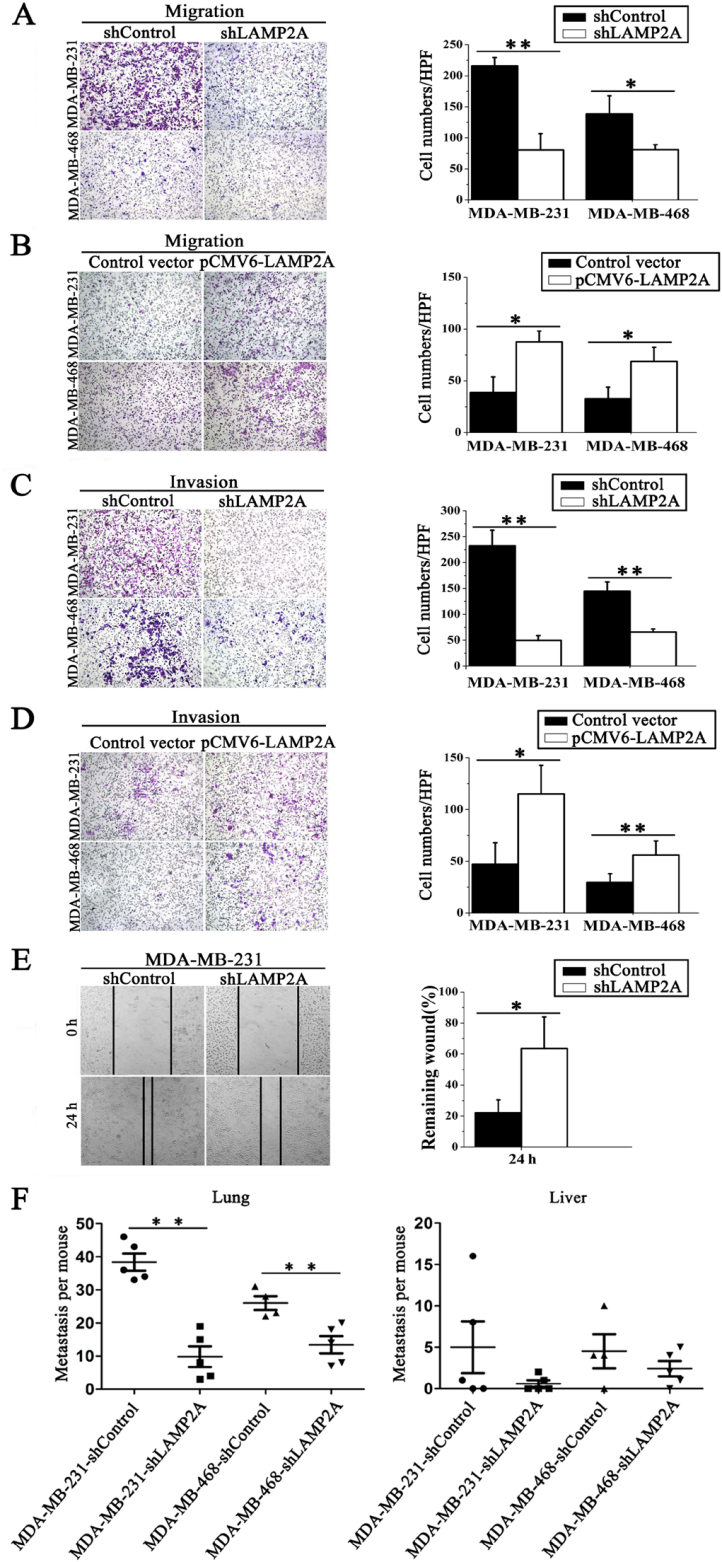
CMA promotes breast cancer cell migration and invasion *in vitro* and *in vivo*. (**A**) and (**B**) The migration of breast cancer cells was detected using a Transwell migration assay. shLAMP2A or shControl MDA-MB-231 and MDA-MB-468 cells (**A**) or transient LAMP2A-overexpressing or control MDA-MB-231 and MDA-MB-468 cells (**B**) were cultured for 6 h, and the cells that migrated to the lower chamber were observed by a microscope after crystal violet staining (left) and counted (right). (**C**) and (**D**) The invasion ability of the two breast cancer cell lines was detected using a Transwell Matrigel invasion assay. shLAMP2A or shControl cells (**C**) and LAMP2A-overexpressing or control cells (**D**) were cultured for 48 h, and cells that invaded into the lower chamber were observed by a microscope after crystal violet staining (left) and counted (right). (**E**) The migration of breast cancer cells was measured by a wound-healing assay. The cells were cultured for 24 h after the wound scratch, and the size of the wound at different times was observed by a microscope (left). The size of the wound was quantified (right). All values are the means ± SD of three different experiments; *P < 0.05, **P < 0.01. (**F**) Nude mice were injected with either shLAMP2A or shControl MDA-MB-231 cells or with shLAMP2A or shControl MDA-MB-468 breast cancer cells via the tail vein. At 60 days after the tail vein injection, the numbers of tumor nodes in the lungs (left) and livers (right) were counted (n = 4 to 5, **P < 0.01, shLAMP2A or shControl).

Cell mobility is a critical marker of metastatic potential in cancer cells^24^. To further ascertain whether CMA activation is implicated in breast cancer cell migration and invasion, we performed classic cell-based Transwell migration assays, Matrigel invasion assays (in which cells had to invade through an extracellular matrix) and wound-healing assays. First, we found that LAMP2A knockdown inhibited the migration and invasion of both MDA-MB-231 and MDA-MB-468 cells towards a gradient (serum) using the Transwell migration and invasion assay (Fig. 3A,C). By contrast, LAMP2A overexpression in MDA-MB-231 and MDA-MB-468 cells promoted cell migration and invasion (Fig. 3B,D). The effect of elevated CMA activity on breast cancer cell migration was also observed with another *LAMP2A* shRNA (shLAMP2A2) in MDA-MB-231 cells, which showed a similar tendency (Supplementary Fig. S2B). To further verify that LAPM2A promotes breast cancer cell metastasis, we performed wound-healing assays. LAMP2A knockdown significantly inhibited the migration ability of MDA-MB-231 cells, causing delayed wound closure when compared to the control cells after 24 h of culture (Fig. 3E). Furthermore, the migration ability of MCF10A cells was also examined, and no significant difference was found between shLAMP2A cells and shControl cells (Supplementary Fig. S3C).

To explore the effect of CMA on metastasis in human breast cancer xenografts, nude mice were injected with shLAMP2A or shControl MDA-MB-231 cells or with shLAMP2A or shControl MDA-MB-468 cells via the tail vein. At 60 days after the tail vein injection, the anatomic distribution of cancer cells in various organs was determined using H&E staining. Surprisingly, we observed multiple metastatic tumor nodules in the lungs of all mice from the control group, and the number of tumor nodules was much lower in mice injected with LAMP2A knockdown cells (~3.8-fold for MAD-MB-231, ~2.0-fold for MAD-MB-468) compared with the control group. This phenomenon was also observed in the liver, although there was no statistical significance between mice injected with shLAMP2A or shControl cells (Supplementary Fig. S4 and Fig. 3F). These data strongly demonstrated that CMA activation promotes breast cancer cell migration and invasion.

### CMA induced the inhibition of macroautophagy activity in breast cancer cells

**Figure 4:**
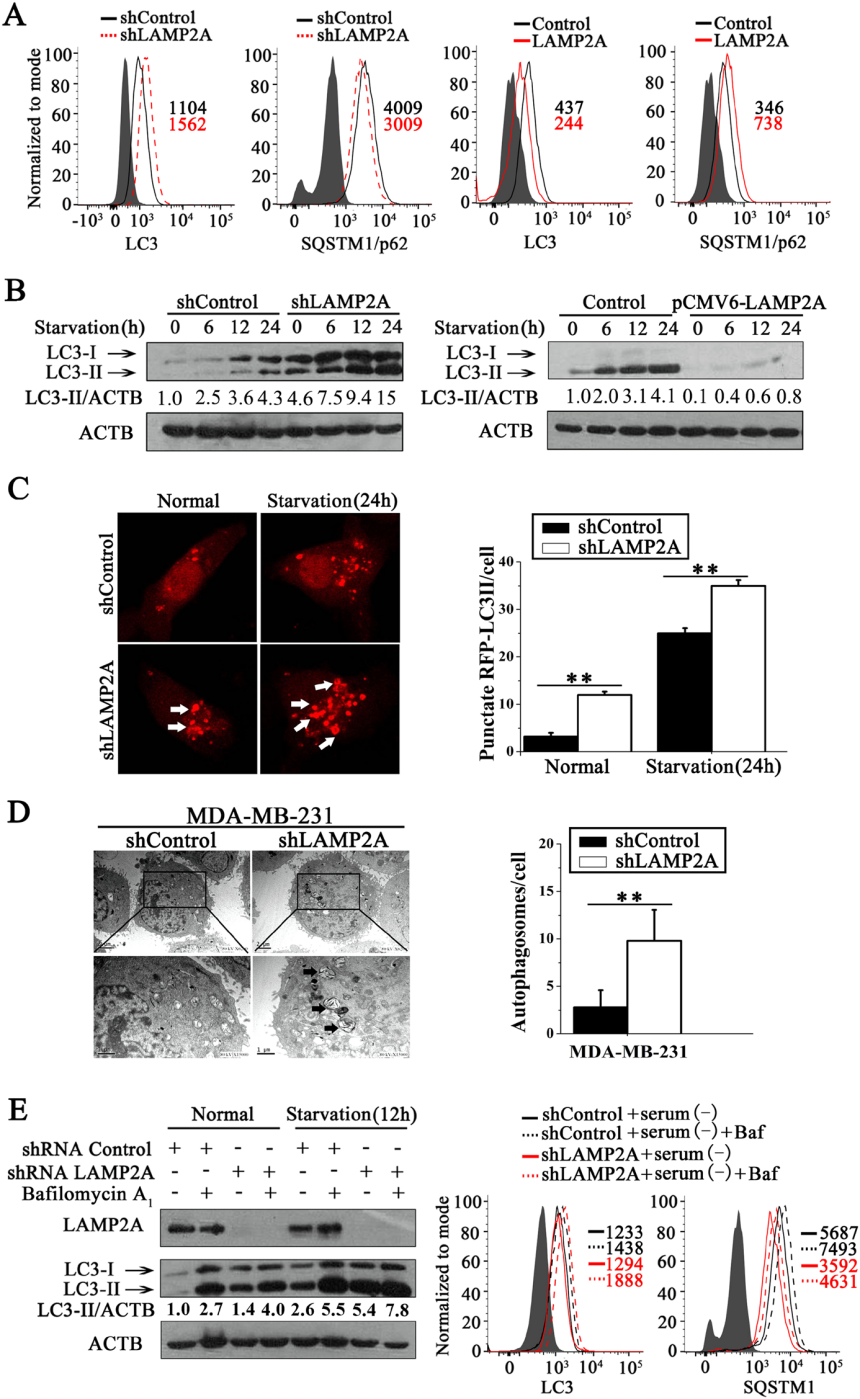
Blockage of CMA increases macroautophagy in breast cancer cells. (**A**) LC3 and SQSTM1 protein levels were detected by flow cytometric analysis in stable LAMP2A knockdown or overexpression cells and related control MDA-MB-231 cells under basal condition. (**B**) Western blotting detected the expression levels of the LC3-II protein in LAMP2A knockdown or overexpression cells and related control MDA-MB-231 cells that were starved for 0, 6, 12 and 24 h. (**C**) The level of macroautophagy in MDA-MB-231 breast cancer cells was monitored at 0 h and 24 h of starvation by observing the RFP-LC3 puncta formation (left). The numbers of RFP-LC3 puncta in each cell was counted (right) (n = 30; **P < 0.01, shLAMP2A versus shControl). Arrows indicate LC3-stained autophagic vacuoles. (**D**) Representative transmission electron microscopic images showed macroautophagic vacuoles in the shLAMP2A or shControl MDA-MB-231 breast cancer cells (left), and autophagosomes were counted (right) (**P < 0.01); arrows indicate autophagosomes. (**E**) The LC3-II and SQSTM1 levels were measured by autophagic flux analysis after bafilomycin A_1_ (Baf, 20 nM) treatment under normal or starvation conditions for 12 h using western blotting (left) and flow cytometric analysis (right). The numbers underneath each lane represent the densitometric quantification of the detected protein after normalizing to β-actin (three independent experiments).

CMA has been shown to induce the inhibition of macroautophagy in mouse embryonic fibroblasts and HeLa cells^15, 17^. Thus, we first explored whether this effect of CMA on macroautophagy also exists in breast cancer cells. The level of macroautophagy was detected by four classical methods. First, under basal condition, flow cytometric analysis showed that LAMP2A knockdown increased the total LC3 level and downregulated the SQSTM1/p62 level in MDA-MB-231 cells, whereas LAMP2A overexpression had the opposite effect (Fig. 4A). Western blotting further indicated that LAMP2A downregulation led to an increased LC3-II amount in MDA-MB-231 cells under starvation conditions, whereas LAMP2A overexpression showed the opposite effect (Fig. 4B). This was also confirmed by the observation of punctate RFP-LC3-positive vesicles using laser confocal imaging (Fig. 4C). Finally, the increased accumulation of autophagosomes was further confirmed in MDA-MB-231 shLAMP2A cells using transmission electron microscopy (Fig. 4D). To exclude the possibility of autophagic flux blockage, LC3-II and SQSTM1 levels, which are both classic markers of autophagic flux, were determined in the presence of the lysosomal protease inhibitor bafilomycin A_1_^25^. As expected, the expression of LC3-II induced by combination of shLAMP2A with bafilomycin A_1_ was higher compared with the expression of LC3-II induced by shLAMP2A or bafilomycin A_1_ treatments alone (Fig. 4E, left). In addition, we also found that bafilomycin A_1_ could increase the accumulation of SQSTM1 under starvation conditions, which was detected by flow cytometric analysis (Fig. 4E, right). The above results indicated that autophagic flux was intact and that the upregulated autophagic response was indeed induced by LAMP2A downregulation.

### CMA-mediated inhibition of macroautophagy is ATG5 dependent

**Figure 5:**
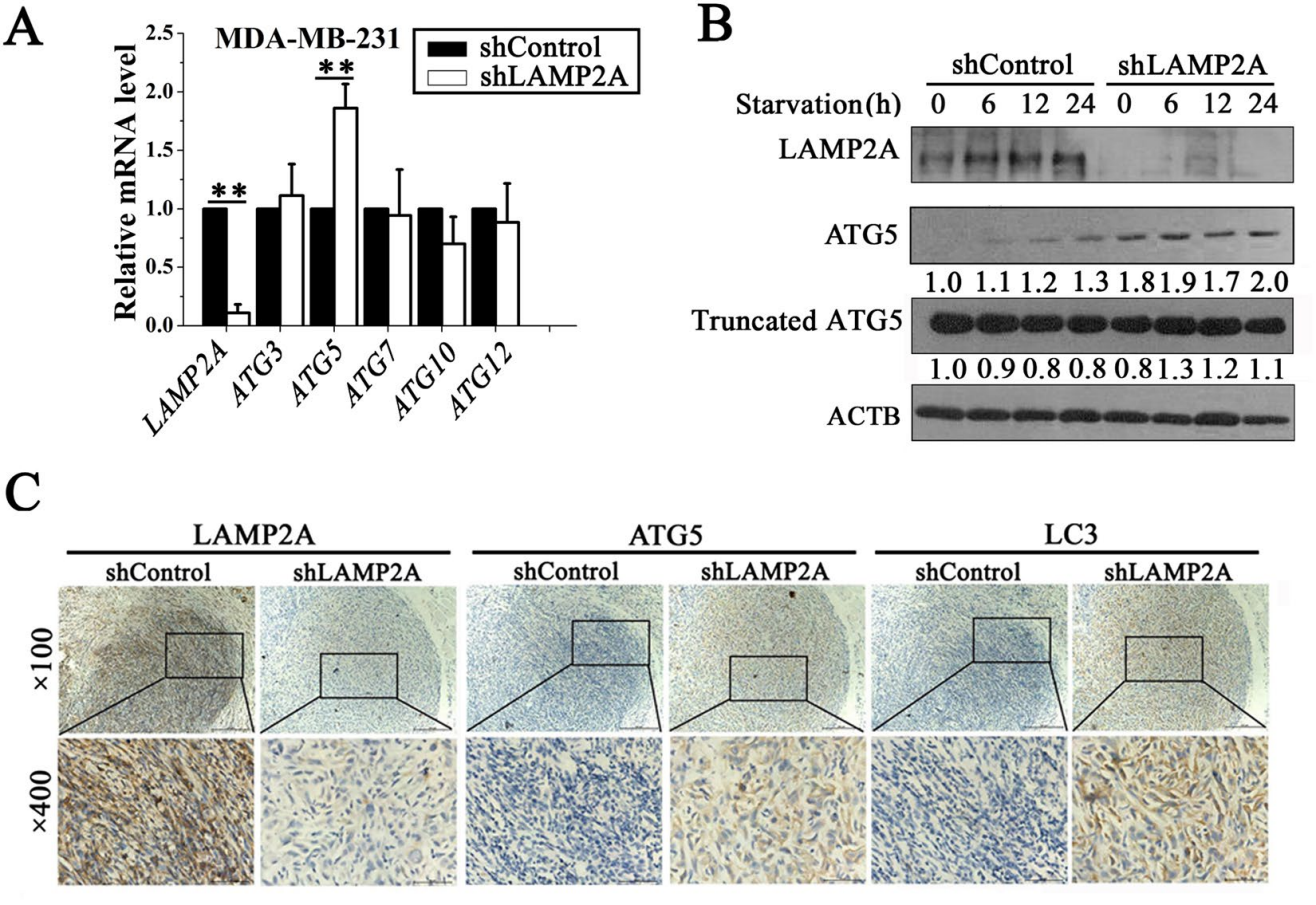
CMA-mediated inhibition of macroautophagy is ATG5 dependent. (**A**) qRT-PCR analysis of the ATG5-dependent macroautophagy gene expression levels in shLAMP2A or shControl MDA-MB-231 cells (left) (n = 3; Student’s t test, **P < 0.01, statistical comparison between the two marked treatment groups). (**B**) shLAMP2A or shControl MDA-MB-231 cells were starved and harvested at the indicated timepoints for immunoblotting of ATG5 and truncated ATG5. The numbers underneath each lane represent the densitometric quantification of the detected protein after normalizing to β-actin. (**C**) LAMP2A, ATG5 and LC3 levels in the subcutaneous tumor tissues of shLAMP2A or shControl MDA-MB-231-implanted mice were measured by immunohistochemistry.

To examine the exact mechanism of CMA-mediated inhibition of macroautophagy, we first observed the effects of CMA on ATG5-dependent macroautophagy, as ATG5 may play a critical role in the crosstalk between CMA and macroautophagy^19^. Through LAMP2A knockdown in MDA-MB-231 cells, we found that LAMP2A inhibition obviously increased *ATG5* mRNA (Fig. 5A) and protein expression (Fig. 5B). In addition, as truncated ATG5 is involved in apoptosis^26, 27^, we also determined its protein levels and found no significant difference in truncated ATG5 levels between LAMP2A knockdown cells and control cells (Fig. 5B).

Furthermore, we also found that the *ATG5* mRNA level was not changed after LAMP2A downregulation in MCF10A cells (Supplementary Fig. S3D). Finally, our *in vivo* experiments showed that LAMP2A knockdown significantly increased LC3 and ATG5 expression in tumors from shLAMP2A MDA-MB-231-implanted mice compared with tumors from shControl MDA-MB-231-implanted mice (Fig. 5C). All of these data suggested that CMA-mediated inhibition of macroautophagy is ATG5-dependent.

### ATG5-dependent macroautophagy inhibition is required for the CMA-mediated growth and metastasis of human breast cancer cells

**Figure 6:**
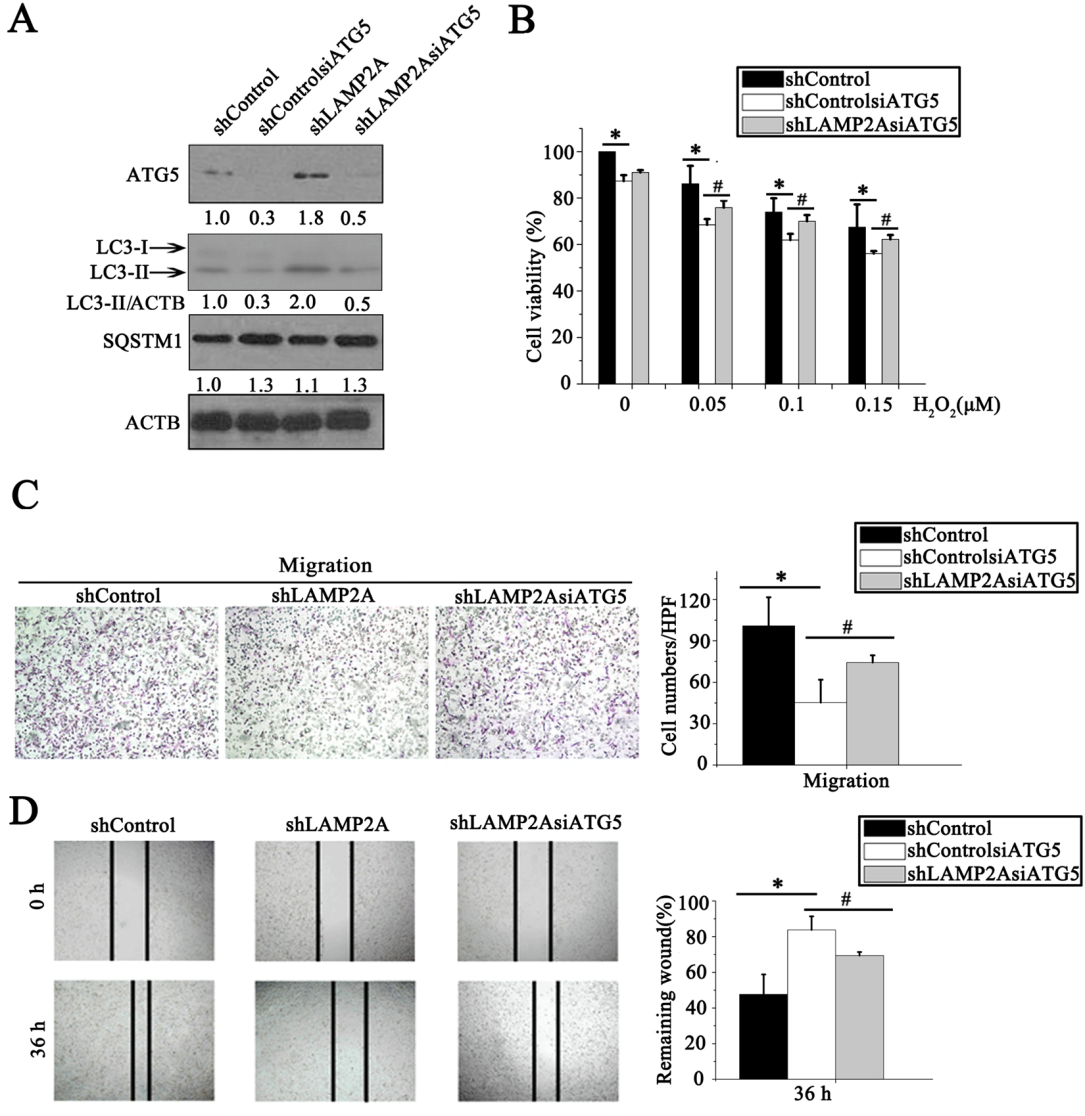
ATG5-dependent macroautophagy inhibition is required for the CMA-mediated growth and metastasis of human breast cancer cells. (**A**) ATG5, SQSTM1 and LC3-II levels were measured by western blotting in shLAMP2A MDA-MB-231 breast cancer cells after transfection with *ATG5* siRNA. (**B**) Cells were treated with different concentrations of H_2_O_2_ for 24 h, and cell viability was measured by an MTT assay. (**C**) The migration of breast cancer cells after transfection with *ATG5* siRNA was detected using a Transwell migration assay. (**D**) The migration of breast cancer cells was measured by a wound-healing assay. The breast cancer cells were transfected with *ATG5* siRNA and cultured for 36 h after the wound scratch, and the size of the wound at different timepoints was observed by a microscope (left). Quantification of the size of the wound (right).

ATG5 knockout or inactivation has been shown to result in increased tumorigenesis in mammalian models^28, 29^. To further evaluate the effect of ATG5-dependent macroautophagy on the CMA-mediated growth and metastasis of human breast cancer cells, we transfected tumor cells with *ATG5* siRNA and evaluated their growth and metastasis. First, we found that inhibition of ATG5 expression reversed the *LAMP2A* RNAi-dependent expression levels of LC3-II and SQSTM1 (Fig. 6A). Moreover, decreased ATG5 expression also reversed the *LAMP2A* RNAi-dependent inhibition of MDA-MB-231 cell growth and survival (Fig. 6B). In addition, we also found that ATG5 knockdown obviously reduced the *LAMP2A* RNAi-dependent inhibition of MDA-MB-231 cell migration in the Transwell migration and wound-healing assays (Fig. 6C,D). These results demonstrated that inhibition of ATG5-dependent macroautophagy is required for the CMA-mediated growth and metastasis of human breast cancer cells.

## Discussion

CMA can selectively degrade specific proteins and help maintain cellular homeostasis under stress conditions, and it is involved in many human diseases^9^. A higher LAMP2A protein level was observed in breast ductal carcinoma tissue of 7 different patients compared with normal adjacent tissue^13^, which indicates that CMA activation has an essential role in breast cancer survival. Due to the high heterogeneity of various cancer cells, we further confirmed this finding that higher LAMP2A protein expression in breast cancer tissues is positively correlated with the malignant progression of breast cancer by assessing LAMP2A expression in 166 breast cancer specimens and 21 normal breast epithelium specimens. In addition, we found that high LAMP2A levels also predict shorter overall survival of breast cancer patients. Our results suggest that LAMP2A may serve as a predictive marker for breast cancer.

Metastasis is the major cause of lethality in cancer patients. Reduced oxygen levels, endoplasmic reticulum stress and nutrient deficiency are the main challenges that breast cancer cells must overcome in the progression of metastasis^30^. Autophagy, which is abnormally activated during cancer cell metastasis, plays an important role in the maintenance of cancer cell viability and promotes cancer cell metastasis under the abovementioned stresses^31^. In this study, we found that CMA activity was associated with breast cancer metastatic carcinoma. The scores for CMA activity (determined by LAMP2A) in the metastatic carcinoma patients were significantly higher compared with the normal tissue group. We also provided evidence from *in vitro* and *in vivo* studies to support this finding. First, silencing LAMP2A significantly inhibited the migration and invasion of breast cancer cells. In addition, upregulation of LAMP2A significantly increased the migration and invasiveness of breast cancer cells. We also found that downregulating CMA activity by LAMP2A knockdown significantly reduced the metastasis of breast cancer cells in xenografts. Thus, our work has demonstrated that CMA promotes breast cancer metastasis both *in vitro* and *in vivo*.

The contradictory roles of macroautophagy in breast cancer are often viewed as confusing. Under nutrient deprivation, hypoxia and therapeutic stress conditions, autophagy seems to function as a protective cell survival mechanism. In addition, the reduced macroautophagy level caused by the downregulation of autophagy genes (e.g., ATG5 and BCN1) significantly inhibits cell death, indicating that the induction of autophagy alone may also be used as a potential therapy. We show here that downregulation of CMA indeed leads to upregulation of macroautophagy in MDA-MB-231 cells and that CMA-mediated downregulation of macroautophagy contributes to breast cancer growth and metastasis *in vitro*. Previous findings have shown that CMA-defective cells such as mouse embryonic fibroblasts maintain normal rates of long-lived protein degradation via upregulated macroautophagy^15^. However, this effect was not observed in lung cancer cells^12^. These discrepancies may result from the high heterogeneity among different cancer types. Future studies are necessary to understand the detailed mechanisms of CMA-mediated suppression of macroautophagy in different cell types and its biological significance and impacts on therapeutic targets. Moreover, future insights into the upstream signaling that leads to CMA activation will provide powerful therapeutic tools to modulate this pathway.

In summary, the repression of ATG5 transcription by elevated CMA is implicated in the downregulation of macroautophagy activity, which further promotes breast cancer cell metastasis. These data highlight novel links among CMA, ATG5-mediated macroautophagy and breast cancer cell metastasis. This study provides a new model for understanding the complex roles of different types of autophagy in tumorigenesis and metastasis.

## Materials and Methods

### Reagents and cell culture

The MDA-MB-231 and MDA-MB-468 human breast cancer cell lines were cultured in RPMI 1640 medium (Gibco) and in Dulbecco’s modified Eagle’s medium (DMEM) (Gibco), respectively. Both were supplemented with 10% fetal bovine serum (Gibco) and incubated in an atmosphere of 5% CO_2_ at 37 °C. Both cell lines were obtained from the cell bank of the Committee on Type Culture Collection of the Chinese Academy of Sciences. More information regarding reagents, constructs and cell culture is presented in Supplemental Materials.

### Tissue arrays and immunohistochemistry

Human breast cancer tissue arrays (BR8010, BR951 and BC8013a) were purchased from Alenabio Biotechnology, and all samples were from Asian patients. Written informed consent was obtained from all subjects before collecting the samples. All the methods were carried out in accordance with the institutional protocols and approved by the Ethics Committee of the Third Military Medical University, Chongqing, China. Immunohistochemistry staining and the IHC score calculation were performed as described previously^32^, detailed in Supplementary Materials and Methods.

### Construction of lentiviral shRNA and overexpression vector and cell infection

The sequences of the small interfering RNAs (siRNAs) targeting the LAMP2A gene were 5′-GCAGTGCAGATGACGACAA-3′ (1283), 5′-GCACCATCATGCTGGATAT-3′ (1383), 5′-CCCAGTGTCATTAGATAAT-3′ (2067) and 5′-GGCTACAACAGAACTTAAA-3′ (3818), defined as shLAMP2A1, shLAMP2A2, shLAMP2A3 and shLAMP2A4, respectively. The hairpin (sense–loop–antisense) for these sequences was inserted in the multicloning region (BamHI and EcoRI) of the pGLV-EGFP vector (GenePharma). For the LAMP2A overexpression vector, the LAMP2A PCR product was obtained by amplifying the LAMP2A sequence from the pCMV6-XL5-LAMP2A vector and cloned into the NotI and BamHI sites of EF1α-LV5-EGFP vector (GenePharma). The primers used were as follows: forward: 5′-GATATGGCGGCCGCGCCACCATGGTGTGCTTCCGCCTCTTCC-3′, reverse: 5′-GTATGGGATCCCTAAAATTGCTCATATCCAGCATGATG-3′. Then, according to the manufacturer’s protocol, HEK293T cells were co-transfected with a shuttle vector (pGLV-EGFP-LAMP2A shRNA or LAMP2A overexpression) and packaging vectors (Helper vector-I, Helper vector-II and Helper vector-III) to generate lentivirus particles. The cellular supernatant was then collected. All of the virus titers reached 2 × 10^8^ TU/ml. MDA-MB-231, MCF10A and MDA-MB-468 cells were infected with lentiviruses expressing the LAMP2A shRNAs or the LAMP2A overexpression vectors (GenePharma), and the culture medium was replaced with fresh medium containing 10% FBS 12 h later. Then, the cells were stably selected by puromycin treatment.

### Association of human recombinant his-GAPDH with lysosomes

Lysosome isolation was performed according to the manufacturer’s instructions using a Lysosome Enrichment Kit for tissue and cultured cells (Pierce, 89839). The association of his-GAPDH with isolated lysosomes was analyzed according to a previous study^33^. Briefly, freshly isolated lysosomes were incubated with protease inhibitor for 10 min on ice. Then, lysosomes (100 μg of protein), 50 μg of his-GAPDH, 2 μg of his-HSC70 and 6 × energy regenerating system (60 mM ATP, 12 mM phosphocreatine, 0.3 mg/ml creatine phosphokinase, 60 mM MgCl_2_, pH 7.3) were mixed together and incubated for 20 min at 37 °C. Lysosomes were collected by centrifugation, washed with PBS, and subjected to SDS-PAGE and immunoblotting for his-GAPDH.

### Western blotting analysis, immunofluorescence staining, quantitative real-time PCR and *in vitro* studies

Western blotting analysis, immunofluorescence staining, quantitative real-time PCR (qRT-PCR) and *in vitro* studies were performed as described previously^20, 32, 34^, detailed in Supplementary Materials and Methods.

### Animal studies

Four- to six-week-old female BALB/c athymic nude mice (Experimental Animal Center of the Third Military Medical University) were housed with free access to food and water for 7 days after arrival. All the animal experiments were carried out in accordance with the institutional protocols and approved by the Ethics Committee of the Third Military Medical University, Chongqing, China. For the tumor growth assay, each mouse was subcutaneously injected in the right flank with 1 × 10^7^ shLAMP2A or shControl MDA-MB-231 cells in 200 µl of serum-free RPMI-1640 medium. The tumor size was measured every 2 days, and the tumor volumes were calculated using the standard formula length × width × width × 0.5. For the metastasis assay, the mice were inoculated with shLAMP2A or shControl MDA-MB-231 cells or with shLAMP2A or shControl MDA-MB-468 cells (2 × 10^6^ cells per mouse in 150 µl RPMI 1640 medium or DMEM) by tail vein injection. After 60 days, the mice were sacrificed. Autopsies were performed, and the subcutaneous tumors were used for immunohistochemistry to detect the macroautophagic level. Organs from the mice of all groups were fixed and routinely stained with H&E to assess the metastases; the numbers of metastases were recorded under a light microscope.

### Statistical analysis

A log-rank test was used for survival analysis, and one-way ANOVA and t-test were used to analyze the variance. Numerical data were expressed as the mean ± SD. All P values < 0.05 were considered significant.

### Data availability statement

All data in our manuscript are available.

## Electronic supplementary material

Supplementary materials
