# Supplementary material for: Downregulation of ATG5-dependent macroautophagy by chaperone-mediated autophagy promotes breast cancer cell metastasis
Source: Sci Rep. 2017 Jul 6;7:4759. doi: 10.1038/s41598-017-04994-x (PMC5500507; doi:10.1038/s41598-017-04994-x)
Supplement: Supplementary file 1 — Supplementary materials [file 41598_2017_4994_MOESM1_ESM.pdf]

Supplementary materials to:

Qi Han, Youcai Deng, Sha Chen, Rui Chen, Mingzhen Yang, Zhujun Zhang, Xiongshan Sun, Wei Wang, Ying He, Fangjie Wang, Xiaodong Pan, Peng Li, Wenjing Lai, Hongqin Luo, Pei Huang, Xiao Guan, Yafei Deng, Jun Yan, Xianjie Xu, Yan Wen, An Chen, Chuanmin Hu, Xiaohui Li* and Shuhui Li*

Downregulation of ATG5-dependent macroautophagy by chaperone-mediated autophagy promotes breast cancer cell metastasis

Supplementary Experimental Procedures:

Antibodies and reagents

The pCMV6-XL5-LAMP2A expression plasmid was purchased from ORIGENE (SC118738), and the RFP-LC3 vector was kindly provided by T Yoshimori at Osaka University. Opti-MEM I (31985-062), Lipofectamine 2000 (11668-027) and puromycin (A11138-03) were from Invitrogen. MTT (M5655), DMSO (D4540) and H_2O_2 (323381) was purchased from Sigma-Aldrich, 1% crystal violet (C0121) was from Beyotime. Antibodies were as follows: LAMP2A (ab18528), HSC70 (ab19136), SQSTM1/P62 (ab56416), truncated ATG5 (ab77580) were from Abcam, ATG5 (2630) and MAP1LC3 (4108) were from Cell Signaling Technology, and β-actin (BM0626), HSP60 (BA1511) were from Boster.

Cell culture

Human normal breast epithelial cell line MCF10A was from Cobioer Biosciences (CBP60419), it was cultured in MEBM medium supplemented with 10% heat-inactivated fetal bovine serum, 20 ng/ml human EGF, 100 ng/ml cholera toxin, 0.01 mg/ml bovine insulin and 500 ng/ml hydrocortisone (all from Cobioer Biosciences). For the starvation experiments, the cells were cultured in complete RPMI 1640 medium without FBS. For bafilomycin A_1 treatment, the cells were incubated with their appropriate medium with bafilomycin A_1 (20 nM) for 12 h.

Tissue array and immunohistochemistry

All the methods were carried out in accordance with the institutional protocols and approved by the Ethics Committee of the Third Military Medical University, Chongqing, China. In total, there are 166 breast cancer specimens and 21 normal breast epithelium specimens for assessing LAMP2A expression, and 50 breast cancer specimens and 7 normal breast epithelium specimens for assessing. The numbers of gradation of the invasive ductal carcinoma were 21, 74 and 28 (Grade I, Grade II and Grade III, respectively) for LAMP2A analysis. Immunohistochemistry staining was performed with an indirect immunoperoxidase method. Briefly, the slides were deparaffinized in xylene, rehydrated in alcohol and water, antigen retrieved, and blocked. Antibodies against LAMP2A (1:100), LC3 (1:100), and ATG5 (1:50) were incubated with the corresponding slides overnight. After treatment with a horseradish peroxidase-labeled polymer, the sections were incubated with DAB (Beyotime, P0203) and counterstained with hematoxylin. Here, the IHC scores were calculated according to the method reported by previous studies[1-2]. For each tumor sample, we determined a proportion score and an intensity score. The proportion score represented the estimated fraction of positive staining tumor cells (0 = none, 1 = < 10%, 2 = 10–50% and 3 = > 50%). The intensity score represented the estimated average staining intensity of positive tumor cells (0 = none; 1 = weak; 2 = intermediate, 3 = strong). The overall amount of LAMP2A and HSC70 protein levels present in each tumor was then expressed as the sum score of the proportion and intensity scores (ranges = 0 and 2 - 6, respectively). We identified TMA scoring > 3 as LAMP2A positive tumors and

TMA scoring < or = 3 as LAMP2A negative tumors[2].

Transfection assay

Transfection of pCMV6-XL5-LAMP2A, RFP-LC3 and *ATG5* siRNA was conducted using Lipofectamine 2000, according to the manufacturer's recommendations, and cells were cultured for 48 h before being harvested.

siRNA sequence

ATG5 siRNA sequence was 5'-GCAACUCUGGAUGGGAUUG-3' (671), and *HIF1A* siRNA sequence was 5'-GGACAAGUCACCACAGGAC-3' (1566)[3]. All siRNA oligos were synthesized by GenePharma.

Lysosome isolation

Briefly, 200 mg of cell pellet (about 4×10^7 cells) was harvested by centrifuging and then suspended in 800 μl of lysosome enrichment buffer A added with protease inhibitors, and then the cell suspension was sonicated at 25 w of the power and 3 s one pulse for 3 bursts. 800 μl of lysosome enrichment buffer B with protease inhibitors was then added and mixed, centrifuged at 500 g for 10 min, the supernatant was collected and applied to a discontinuous density gradient (30%, 27%, 23%, 20% and 17% with Optiprep media). Then the samples were ultracentrifuged at 140000 g for 2 hours at 4°C, the top band of the gradient was carefully collected as the lysosomes. Then the lysosomes were mixed with 2-3 volumes of PBS and centrifuged at 18000 g for 30 min at 4°C. The lysosomal pellet integrity was verified by measuring the activity of β-hexosaminidase, a lysosomal enzyme, in the incubation medium[4], and β-hexosaminidase ELISA kit was from ShangHai Qiaodu Biotechnology (QD30352).

Expression and purification of human GAPDH and HSC70

cDNAs of human *GAPDH* and *HSC70*[5] were cloned into the NdeI and XhoI sites of pet22b vector after codon partial tropism transformation. The PCR primers used for amplifying *GAPDH* and *HSC70* cDNA were as follows: *GAPDH* forward: 5′-GTGACCATATGGGTAAAGTCAAAGTAGGT-3′, *GAPDH* reverse: 5′-GTGACCTCGAGTTCTTTGGACGCCATGTGC-3′, *HSC70* forward: 5′-GACACCATATGAGCAAAGGCCCT-3′, *HSC70* reverse: 5′-GTGTCCTCGAGGTCAACTTCTTCAATGGTCGGGCCGGAG-3′. Bacteria strains BL21 (DE3) carrying the pet22b-his-HSC70 and pet22b-his-GAPDH plasmid were induced expression by 0.3 mM of IPTG and incubated for an additional 12 h at 20 °C with 100 μg/ml ampicillin. Then, bacteria were collected by centrifugation (10000 g for 5 min) and washed by PBS, then the pellet was resuspended with 50 mM Tris-HCl, 2 mM EDTA, 100 mM NaCl (pH 8.0), and sonicated at 400 w of the power and 10 s one pulse for 40 bursts. After centrifuged at 10000 g for 30 min, the supernatant was collected and commonly purified by Nickel column.

Western blotting analysis

The cells were harvested and lysates were prepared with RIPA buffer (Beyotime, P0013B). Protein concentrations were determined with the BCA kit (Beyotime, P0011). Then, the proteins (30 μg/well) were subjected to electrophoresis on a 8-12% SDS-PAGE gel, transferred onto polyvinylidene fluoride membranes (Bio-Rad, 162-0177), blocked with 5% bovine serum albumin (BSA) and incubated with the corresponding primary antibodies at 4°C overnight. The membranes were washed and incubated with secondary antibodies and then detected by ECL kit (Millipore, WBKLS0500).

Immunofluorescence staining

Cells grown on sterile cover slips were stained with 25 nM LysoTracker red (Invitrogen, L12492), a lysosomal marker, for 30 min and washed with 1 × PBS followed by fixation with 4% paraformaldehyde for 15 min and then were blocked with goat serum for 30 minutes and incubated with the primary antibody rabbit IgG anti-LAMP2A (1:150) at 4°C overnight. The slides were then extensively washed with PBS and incubated with the fluorescent secondary antibody (Beyotime, P0179) for one hour. Finally, the slides were washed with PBS and visualized using a fluorescence microscope (Leica microsystems); the CMA activity was detected by investigating the distance between LAMP2A-positive lysosomes and the nucleus.

qRT-PCR Analysis

The total RNA was extracted from cultured cells using Trizol (Invitrogen, 15596-018) and was used for reverse transcription with ReverTra Ace-α-kits (TOYOBO, FSK-100). qRT-PCR was performed using the SYBR green PCR master mix (TIANGEN, FP201-02), according to the manufacturer's instructions.

LAMP2A forward: 5′- GTGCAACAAAGAGCAGACTGT-3′
LAMP2A reverse: 5′-CCGCTATGGGCACAAGGAA-3′
LAMP2B forward: 5′-AATGGCTCCGTTTTCAGCAT-3′
LAMP2B reverse: 5′-GGTGTCATCATCCAGCGAAC-3′
ATG3 forward: 5′-CTGATGCTGGCGGTGAAGATG-3′
ATG3 reverse: 5′-GGAGGTGGTGGCAGATGAGG-3′
ATG5 forward: 5′-ATGACAGATGACAAAGATG-3′
ATG5 reverse: 5′-CTCATAACCTTCTGAAAGTG-3′
ATG7 forward: 5′-GGAACAAGCAGCAAATGAGATATGG-3′
ATG7 reverse: 5′-GGAAGACAGAGGGCAGGATAGC-3′
ATG10 forward: 5′-GACACTATTACGCAACAGGAACATC-3′
ATG10 reverse: 5′-GGCATAACTCAGAGGTAGATTCAGC-3′
ATG12 forward: 5′-CATTGACCTGCTGGCTGAATACC-3′
ATG12 reverse: 5′-TCTGTCCTATGTGCTTGCTCTCC-3′

MTT assay

For cell growth, the MDA-MB-231 cells (1.5 × 10^3/well), MDA-MB-468 cells (3 × 10^3/well) and MCF10A cells (3 × 10^3/well) were seeded in 96-well plates and

cultured for 12 h for adhesion. The number of viable cells was determined using the MTT assay. The cell numbers were counted after an additional 0, 1, 2, 3, 4, 5, 6, and 7 days, respectively. For H_2O_2 treatment, the cells were treated with different concentrations (0, 0.05, 0.1 and 0.15 μM) of H_2O_2 for 24 h. Then, 20 μl of the MTT solution (5 mg/ml in PBS) was added to each well, and incubated for 4-6 h at 37°C. The solution was discarded and 200 μl DMSO was added to each well. After gently rocking for 10 min, the plates were detected at 490 nm.

Colony formation assay

The cells were seeded (300 cells/well) onto 6-well plates and allowed to form colonies for two weeks. Then, the colonies were fixed with 4% paraformaldehyde and stained with 1% crystal violet. After rinsing, the colonies were counted under a microscope.

Migration and invasion assay

For the migration assay, the cells were directly seeded in a transwell chamber (Millipore, PIEP12R48) with 8-μm pores in the membranes and cultured for 6 h in a 24-well plate. For the *LAMP2A* shRNA treatment, cells were seeded at 5×10^4/well; for the LAMP2A overexpression and *ATG5* siRNA treatment, the cells were seeded at 2×10^4/well; for MCF10A, the cells were seeded at 6.5×10^4/well. For the invasion assay, the upper chamber was coated with 40 μl extracellular matrix gel (Sigma, E0282) that was diluted 1:2 with cold DMEM medium and incubated 37°C for 30 min to form the ECM gel. The cells were seeded on the Matrigel and cultured for 48 h. The upper chamber was supplied with serum-free medium to allow the cells to migrate or invade towards the complete growth medium. Then, the cells in the upper chamber were wiped off and the cells on the lower surface were fixed and stained with 1% crystal violet before mounting on glass slides. Finally, the cells in five randomly chosen fields were counted under a microscope.

Transmission electron microscope

The cells (2×10^6) were seeded in 10-cm dishes and allowed to attach overnight. Then, the cells were collected and fixed in ice-cold 2.5% glutaraldehyde in 0.1 M PBS, rinsed with PBS, postfixed in 1% OsO_4 with 0.1% potassium ferricyanide, washed again, dehydrated with graded alcohol, and embedded in Epon. Semithin sections (300 nm) were cut using a Reichart Ultracut (Leica). Then, the sections were counterstained with Reynold’s lead citrate and examined under a JEOL 1210 transmission electron microscope.

Monitoring autophagy by flow cytometric analysis

Cytofix/Cytoperm™ Fixation/Permeabilization Kit was from Becton& Dickinson Company (554714), LC3B (PE Conjugate) antibody was from Cell Signaling Technology (8899). Briefly, 5×10^5 cells were fixed and permeabilized by Fixation/Permeabilization solution for 20 minutes in dark at 4°C, washed for two

times and incubated with negative control, LC3B or SQSTM1 antibodies at 4°C for 30 minutes in the dark. After washed two times, cells were resuspended in Staining Buffer and followed by flowcytometric analysis.

Supplementary References

1 Allred, D. C. *et al.* Association of p53 protein expression with tumor cell proliferation rate and clinical outcome in node-negative breast cancer. *J Natl Cancer Inst* 85, 200-206 (1993).

2 Zhang, Z. *et al.* IBP regulates epithelial-to-mesenchymal transition and the motility of breast cancer cells via Rac1, RhoA and Cdc42 signaling pathways. *Oncogene* 33, 3374-3382, doi: 10.1038/onc.2013.337 (2014).

3 Rouschop, K. M. *et al.* The unfolded protein response protects human tumor cells during hypoxia through regulation of the autophagy genes MAP1LC3B and ATG5. *J Clin Invest* 120, 127-141, doi:10.1172/JCI40027 (2010).

4 Qi, L. *et al.* The role of chaperone-mediated autophagy in huntingtin degradation. *PLoS One* 7, e46834, doi:10.1371/journal.pone.0046834 (2012).

5 Dworniczak, B. & Mirault, M. E. Structure and expression of a human gene coding for a 71 kd heat shock 'cognate' protein. *Nucleic Acids Res* 15, 5181-5197 (1987).

Supplemental Figures and Figure Legends:

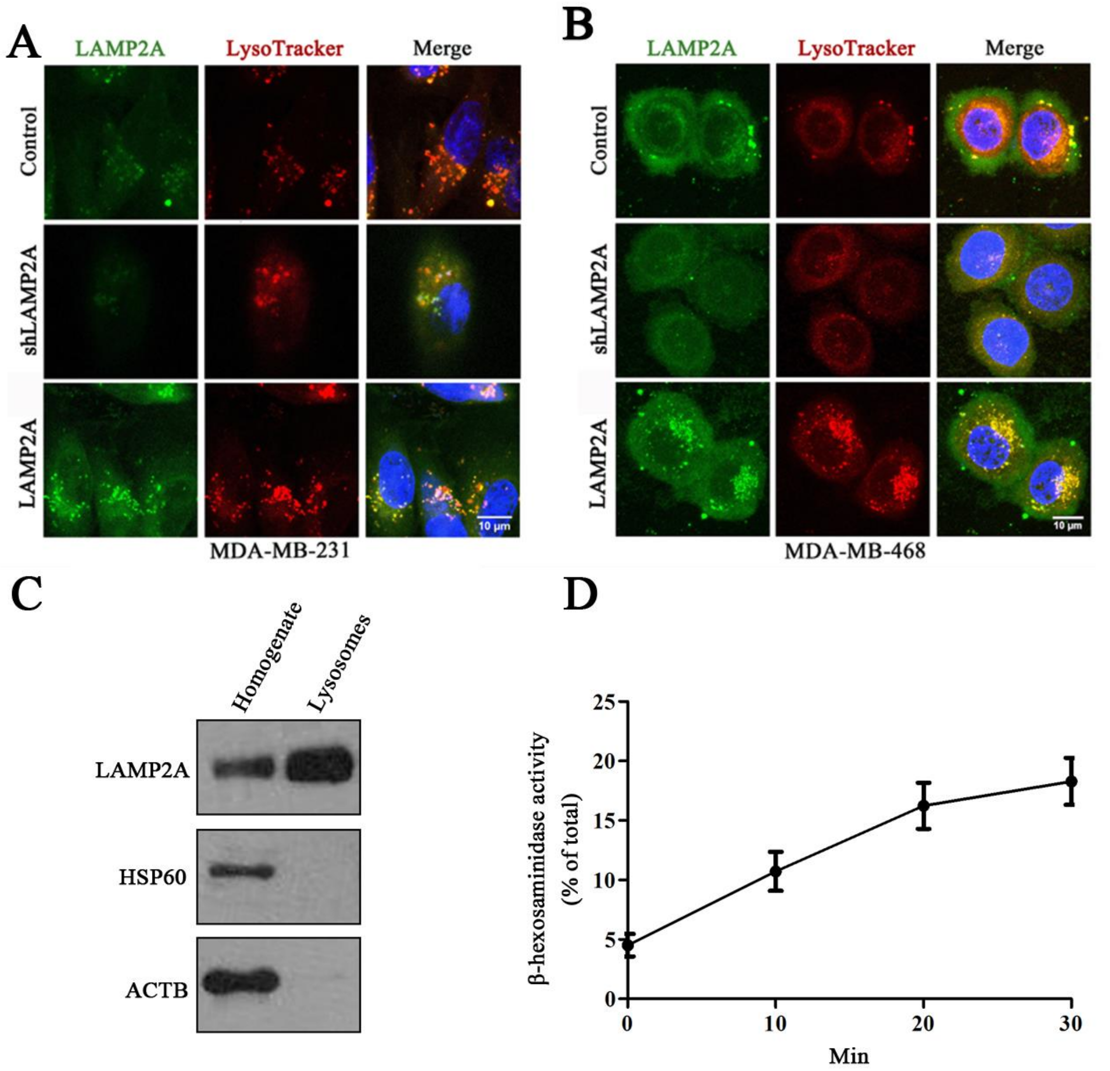

Figure S1. LAMP2A knockdown or overexpression downregulated or increased CMA activity. (A) and (B) CMA activity was detected by the distance of LAMP2A-positive lysosomes to the nucleus. Representative LAMP2A (green fluorescence, left), LysoTracker (red fluorescence, middle) and merged images (right) of breast cancer cells after LAMP2A knockdown or overexpression. (C) The purity of the isolated lysosomes was detected by lysosomes marker LAMP2A, cytoplasm marker β-actin and mitochondrial marker HSP60. (D) The integrity of the isolated lysosomes was determined by β-hexosaminidase. Release of all lysosomal was induced by incubation with 0.1% Triton X-100.

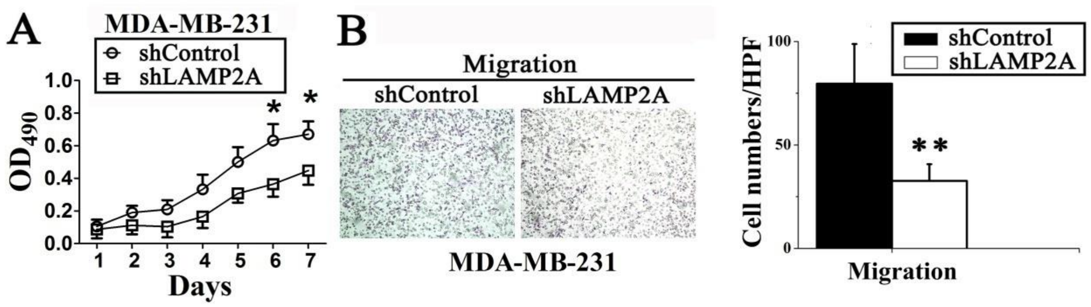

Figure S2. CMA promotes breast cancer cell growth and migration. (A) and (B) LAMP2A was knocked down by LAMP2A shRNA2 in MDA-MB-231 cells, and cell growth rate was determined by the MTT assay at indicated timepoints (A); migration ability was determined using transwellmigration assay (B). n = 3; student's t test, *P < 0.05 and **P < 0.01, shLAMP2A versus shControl at the same timepoint.

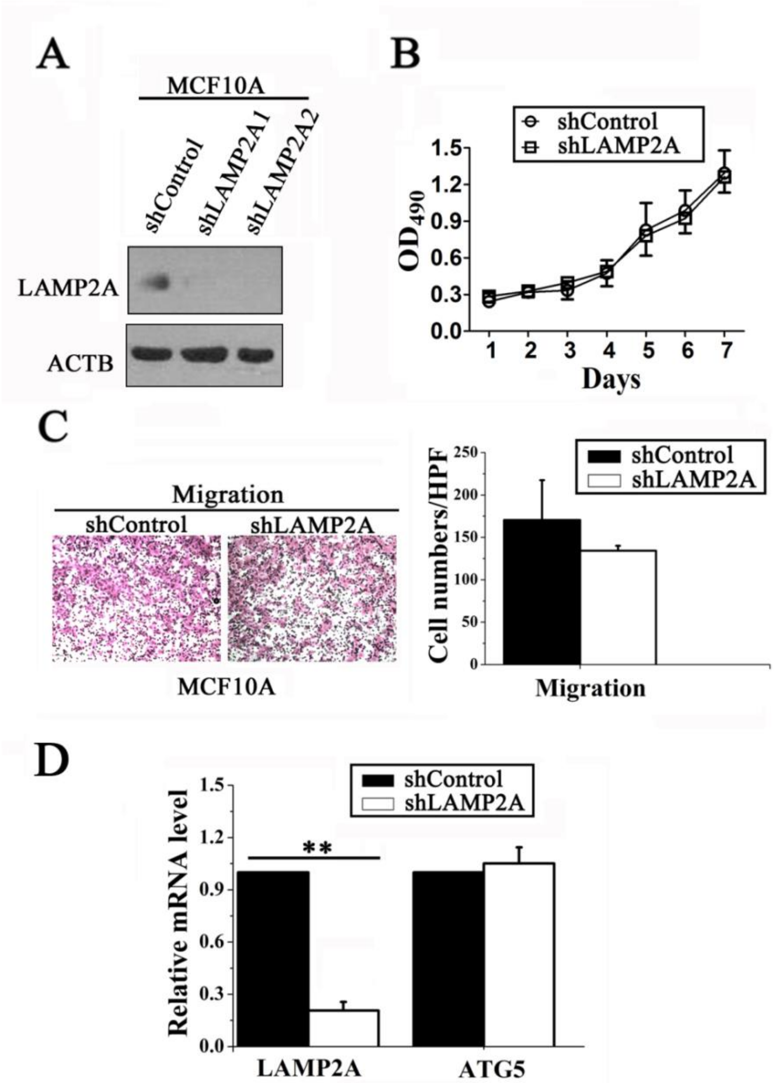

Figure S3. Downregulation of LAMP2A has no effect on cell growth and

migration in normal breast epithelial cells. (A) The stable inhibitory efficiency of shRNAs against LAMP2A in MCF10A cells was determined by immunoblotting. (B) The cell growth rate was determined by the MTT assay at indicated timepoint in MCF10A cells. (C) The migration of MCF10A cells was detected using transwell migration assay. (D) The *ATG5* gene expression level in MCF10A cells was detected after LAMP2A knockdown with qRT-PCR analysis. ***P*< 0.01 indicatesstatistical comparison between the two marked treatment groups. n = 3 independent cultures for each experiment.

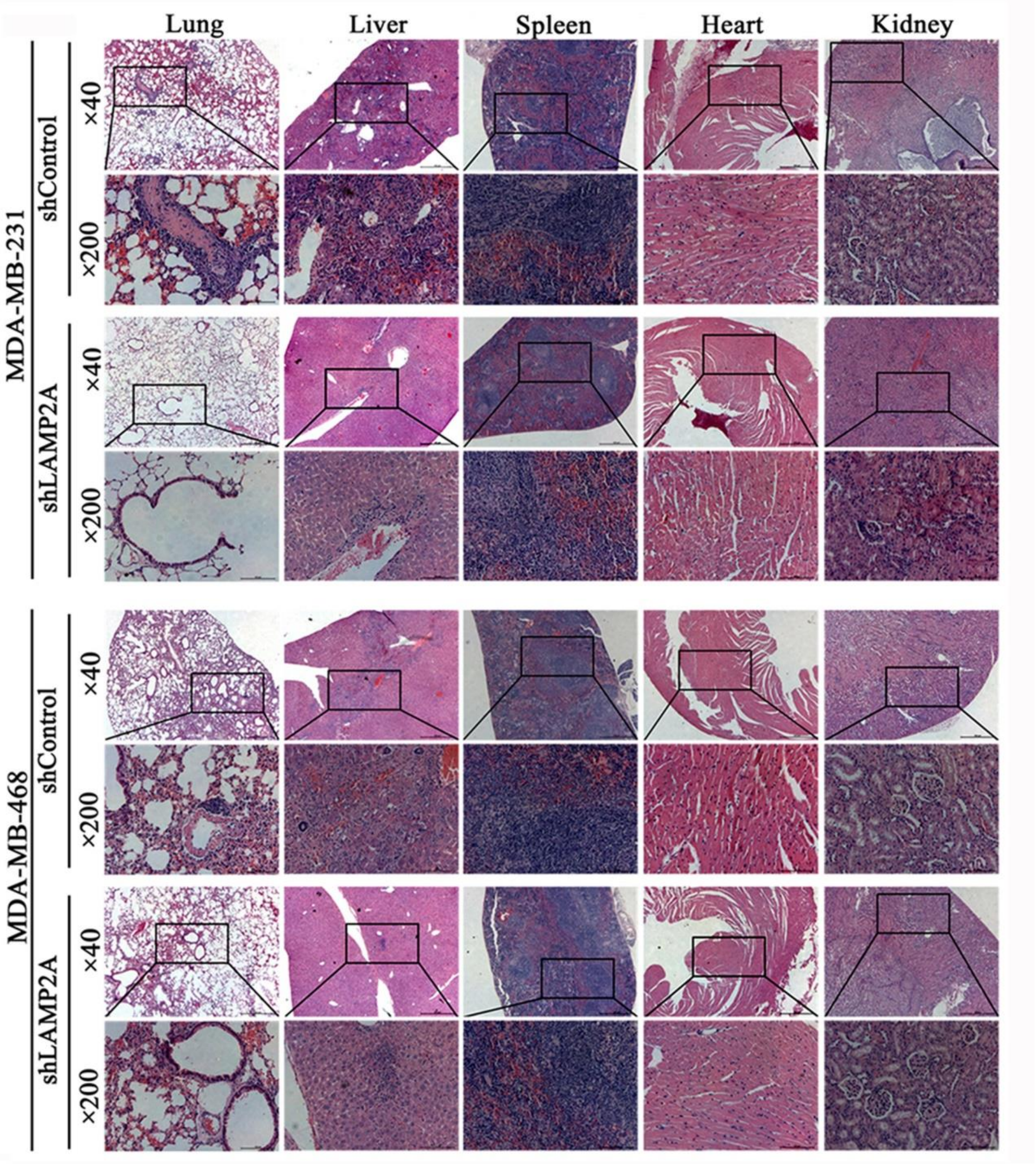

Figure S4. CMA is required for tumor metastasis *in vivo*. Nude mice were injected with either shLAMP2A or shControl MDA-MB-231 or with shLAMP2A or shControl MDA-MB-468 breast cancer cells via the tail vein. At 60 days after the tail vein injection, anatomic distribution of cancer cells in various organs was determined using hematoxylin and eosin (H&E) staining.
